# A Multi-centre, Single Arm, Non-randomized, Prospective European Trial to Evaluate the Safety and Efficacy of the HistoSonics System in the Treatment of Primary and Metastatic Liver Cancers (#HOPE4LIVER)

**DOI:** 10.1007/s00270-022-03309-6

**Published:** 2022-11-15

**Authors:** Tze Min Wah, Maciej Pech, Maximilian Thormann, Xavier Serres, Peter Littler, Benjamin Stenberg, James Lenton, Jonathan Smith, Philipp Wiggermann, Mathis Planert, Joan Vidal-Jove, Guido Torzilli, Luigi Solbiati

**Affiliations:** 1 Department of Diagnostic and Interventional Radiology, Institute of Oncology, St. James’s University Hospital, Leeds Teaching Hospitals NHS Trust, Leeds, LS9 7TF UK; 2 Department of Interventional Radiology, Universitätsklinikum Magdeburg, Magdeburg, Germany; 3 Clinic for Radiology and Nuclear Medicine, University Hospital Magdeburg, Magdeburg, Germany; 4 Department of Interventional Radiology, Hospital Universitari Vall d’Hebron, Barcelona, Spain; 5 Department of Interventional Radiology, Freeman Hospital, Newcastle Upon Tyne, UK; 6 Department of Interventional Radiology, Städtisches Klinikum Braunschweig, Brunswick, Germany; 7 Department of Surgical Oncology, Institut Khuab for Interventional Oncology, Barcelona, Spain; 8 Department of Hepatobiliary & General Surgery, Humanitas University Hospital, Milan, Italy; 9 Department of Biomedical Sciences, Humanitas University, Pieve Emanuele, Milan, Italy; 10 IRCCS Humanitas Research Hospital, Rozzano, Milan, Italy; 11 Leeds Institute of Medical Research (LIMR), University of Leeds, Leeds, UK

**Keywords:** Image-guided ablation, Ultrasound, Histotripsy, Non-thermal ablation, Liver cancer, Hepatocellular carcinoma, Colorectal liver metastases, Technical success, Complications

## Abstract

**Purpose:**

Image-guided thermal ablation are established treatment options for non-surgical patients with primary and metastatic liver cancers. However, there are limitations with nonuniformity of cancer tissue destruction, heat sink effect and the risk of thermal ablative injury. The current non-thermal ablative techniques have high risk of local recurrence and are not widely adopted. Histotripsy is a treatment technology that destroys targeted tissue under ultrasound visualization via mechanical destruction through the precise application of acoustic cavitation and can offer the potential of non-invasive, non-thermal and non-ionizing radiation cancer treatment. The aim of this multi-centre non-randomized phase I/II trial is to assess the initial safety and efficacy of the prototype investigational ‘System’ in the treatment of primary and metastatic liver cancers.

**Methods/Design:**

All non-surgical patients with primary/metastatic liver cancers having had previous liver directed therapy, radiation therapy or image-guided ablation may be offered image-guided Histotripsy as per trial protocol. The co-primary endpoints are technical success and procedural safety. Technical success is determined, at ≤ 36 h post procedure, by evaluating the histotripsy treatment size and coverage. The procedural safety is defined by procedure related major complications, defined as Common Terminology Criteria for Adverse Events (CTCAE version 5) grade 3 or higher toxicities, up to 30 days post procedure. This phase I/II trial has intended to recruit up to 45 patients to show safety and efficacy of image-guided histotripsy in liver cancers.

**Trail Registration:**

Clinicaltrials.gov identifier-NCT04573881; NIHR CRN CPMS-ID 47572.

## Introduction

In the last two decades, with the increased cancer incidence and greater cancer detection at earlier stage has prompted the need for minimally invasive cancer treatments. Since 2000, Interventional Oncology (IO) has emerged as a clinical discipline to compliment surgical, clinical and medical oncology to provide minimally invasive cancer treatments with image-guided ablation using thermal and non-thermal technologies [1, 2].

For early-stage hepatocellular carcinoma (HCC), image-guided thermal ablation is an established part of the treatment algorithm in the BCLC guidelines [3]. Additionally, there are a few randomized controlled trials that are currently ongoing to address the level 1 evidence gap in the literature specifically for colorectal liver metastases (CRLM) [4, 5]. In a multi-disciplinary cancer board, image-guided ablation can often be the alternatives for those patients who are not eligible for surgical resection.

Current image-guided ablation technology consists of both thermal and non-thermal ablative methods. The thermal ablation technologies have included radiofrequency ablation (RFA), microwave ablation (MWA), cryoablation (CRYO) and high-intensity focused ultrasound (HIFU), whilst irreversible electroporation (IRE) is the only more widely adopted non-thermal ablation technique in cancer treatment [6–9]. Image-guided thermal ablation with RFA and MWA is established treatment options for non-surgical patients with primary and metastatic liver cancers. However, there are unmet clinical needs such as nonuniformity of cancer tissue destruction, heat sink effect and the risk of thermal ablative injury [10–13]. The current non-thermal ablative techniques with and irreversible electroporation (IRE) have high risk of local recurrence and are not widely adopted [14, 15].

Recent research in cavitation-based focused ultrasound (histotripsy) is a promising option to destroy liver cancers and offers the potential to overcome the limitations of current ablative technologies [16, 17]. The preclinical study has shown successful liver tumour ablation in the targeted volume in the in vivo porcine model [18, 19]. In addition, studies evaluated the safety of trans-costal histotripsy where ribs are highly reflective and absorbent of ultrasound waves and shown no damage to ribs or tissues using the trans-costal approach [20, 21]. In 2019, Longo et al. demonstrated the safety and feasibility of using robotic assisted sonic therapy (RAST) technique using a modified ultrasound pulse sequence to treat liver in a porcine model that mitigated body wall injuries [22].

Histotripsy is a therapeutic ultrasound technology that uses high power and low frequency ultrasound energy, and applied mechanical bioeffect to liquefy tissue into acellular debris without thermal effect to the surrounding tissue, In contrast to conventional HIFU treatment where it uses the high power and high frequency ultrasound energy to deliver thermal bioeffect to the target tissue [23]. The advantages of mechanical histotripsy, when compared to the conventional ablative technologies, are the non-invasive ‘needle-less’ approach in cancer treatment without the need for ablative electrode insertion, precision in targeting with ultrasound visualization, without radiation or the collateral damage from thermal effect and provides real-time feedback from ultrasound imaging for pre- and peri-operative treatment planning as well as monitoring [16, 18, 24, 25]. Therefore, image-guided histotripsy potentially offers an important clinical advancement for liver cancer treatment and the translational research in patients would be crucial to study its safety and efficacy.

The First in Human study was conducted in Barcelona (NCT03741088), 11 non-curable multifocal liver tumours were treated with the clinical prototype device manufactured by Histosonics, Inc (VORTX Rx device) shown successful predictable liver tumour volume ablation without any significant device-related side effects [26] and this has provided the evidence to progress to a multi-centre trial in Europe (NCT04573881). The aim of this multi-centre non-randomized phase I/II trial is to assess the safety and efficacy of the prototype investigational ‘System’ in the treatment of primary and metastatic liver cancers to support medical device regulatory approval.

## Methods and Design

Study methods are reported with reference to Standard Protocol Items: Recommendations for Interventional Trials Checklist (SPIRIT) [27].

This trial is a Clinical Trial of an Investigational Medicinal Products (CTIMP) trial registered on clinicaltrials.gov (NCT04573881) [28] and also adopted onto National Institute of Health Research (NIHR) portfolio studies (NIHR CRN CPMS-ID 47572). The trial is funded and sponsored by HistoSonics, Inc. (Plymouth, MN, USA) and it is administered by IQVIA Med Tech (GENAE, associates, an IQVIA business, Belgium), an independent Contract Research Organisation (CRO) which facilitated the trial sites monitoring as per Good Clinical Practice (GCP). The trial is overseen by Data Safety Monitoring Board and Clinical Events Committee as part of the trial steering committee (TSC) consisted of a team of independent scientific and clinical experts. The patient’s clinical data is uploaded using electronic case report form (eCRF), and all the trial patients’ imaging assessments are uploaded and managed by Intrinsic Imaging LLC (Bolton, MA, USA), an independent imaging core lab.

The trial protocol is designed to meet the requirements of the initial medical device regulatory approval for regulatory approval for the destruction of liver tissue. It is the first multi-centre, single arm, non-randomized, prospective trial to evaluate the safety and efficacy of the prototype ‘System’ in the treatment of primary and metastatic liver cancers. All the liver cancers must be visualized with ultrasound to be targeted. For primary liver cancer, the diagnosis of hepatocellular carcinoma (HCC) on imaging must meet United Network for Organ Sharing and Organ Procurement and Transplantation Network (UNOS-OPTN) class 5 diagnostic criteria for HCC [29] or the LIRADS [30] diagnostic criteria for HCC, and pathology proven HCC. The imaging diagnostic criteria of LR-5 are equivalent to class 5 using the OPTN-UNOS system.

### Inclusion Criteria [28]

1. Patient is ≥ 18 years of age
2. Patient has signed the Ethics Committee (EC) or Institutional Review Board (IRB) approved trial Informed Consent Form (ICF) prior to any trial related tests/procedures and is willing to comply with trial procedures and required follow-up assessments
3. Patient is diagnosed with hepatocellular carcinoma (HCC) or liver metastases from other primary cancers · Subject that is a HCC patient must have a targetable lesion(s) i.e. lesions that are visualized with ultrasound. These lesions must meet the United Network for Organ Sharing and Organ Procurement and Transplantation Network (UNOS-OPTN) class 5 diagnostic criteria for HCC [29]. Patient that is diagnosed with liver metastases must have prior diagnosis of primary tumour or metastatic tumour to identify the primary cancer type. Patients must have untreated new or growing liver tumour(s) radiologically consistent with metastases. Note: A biopsy is required to confirm metastatic disease and the pathological results must be obtained prior to the procedure, does not need to be the targeted tumour(s)
4. Patient is able to undergo general anaesthesia
5. Patient has a Child–Pugh Score of A or B (up to B8)
6. Patient has an Eastern Cooperative Oncology Group Performance Status (ECOG PS) grade 0–2 at baseline screening
7. Patient meets the following functional criteria, ≤ 7 days prior to the planned procedure date:
  - Liver function: Alanine transaminase (ALT) and Aspartate transaminase (AST) < 2.5 × Upper Limit Normal (ULN) and/ Bilirubin < 2 × ULN
  - Renal function: serum creatinine < 2 × ULN
  - Hematologic function: neutrophil count > 1.0 × 10^9/L and platelet > 50 × 10^9/L
8. Patient has an International Normalized Ratio (INR) score of < 2.0, ≤ 7 days prior to the planned procedure date
9. Patient has not responded to and/or has relapsed and/or is intolerant of other available therapies including locoregional therapies, chemotherapy, immunotherapy and targeted therapies.
10. The tumour(s) selected for histotripsy treatment must be ≤ 3 cm in longest diameter
11. Patient has an adequate acoustic window to visualize targeted tumour(s) using ultrasound imaging
12. Patient has a maximum of three (3) tumours to be treated with histotripsy during the procedure, regardless of how many tumours the subject has.

Note: If the patient is treated with surgical resection prior to the procedure, the resection must be performed ≥ 2 weeks prior to the planned procedure date.

### Exclusion Criteria [28]

1. Patient is pregnant or planning to become pregnant or nursing (lactating) during the trial period
2. Patient is enrolled in another investigational trial and/or is taking investigational medication or treated with an investigational device ≤ 30-days prior to planned procedure date
3. In the Investigator’s opinion, the patient has co-morbid disease(s) or condition(s) that would cause undue risk and preclude safe use of the HistoSonics System
4. Patient has a serum creatinine > 2.0 mg/dL or estimated glomerular filtration rate (EGFR) < 30, unless already on dialysis
5. Patient has major surgical procedure or significant traumatic injury ≤ 2 weeks prior to the planned procedure or not fully recovered (CTCAE grade 1 or better)[31] from side effects/complications of such procedure or trauma
6. Patient has not recovered to Common Terminology Criteria for Adverse Events (CTCAE) [31] grade 1 or better from any adverse effects (except alopecia, fatigue, nausea, vomiting and peripheral neuropathy) related to previous anti-cancer therapy
7. Patient has a history of bleeding disorders (e.g. von Willebrand disease) or suspected to have, bleeding disorders that are un-correctable
8. Patient has un-correctable coagulopathy
9. Patient has a planned cancer treatment (e.g. resection, chemotherapy, etc.) after the planned procedure date and prior to completion of the 30-day follow-up visit
10. Patient has previous treatment with bevacizumab that has not been discontinued > 40 days prior to the planned procedure date
11. Patient has planned bevacizumab treatment prior to completion of the 30-day follow-up visit
12. Patient has previous treatments with chemotherapy and/or radiotherapy that has not been discontinued ≥ 2 weeks prior to the planned procedure date and has not recovered (CTCAE grade 1 or better)[31] from related toxicity
13. Patient has previous treatment with immunotherapies that has not been discontinued ≥ 4 weeks prior to the planned procedure date or has not recovered from related toxicity (CTCAE grade 1 or better)[31]
14. Patient has a life expectancy less than six (< 6) months
15. In the opinion of the Investigator, histotripsy is not a treatment option for the patient
16. Patient has a concurrent condition that, in the investigator’s opinion, could jeopardize the safety of the subject or compliance with the protocol
17. Patients’ tumour(s) is not treatable by the System’s working ranges (refer to User Manual that is provided for the trial participating site).
18. Patient has a known sensitivity to contrast media and cannot be adequately premedicated
19. Patients’ targeted tumour(s) has/have had prior locoregional therapy (e.g., ablation, embolization, radiation)
20. Patient with tumour that is eligible for surgical resection will be excluded from histotripsy enrolment
21. Targeted tumour(s) treatment volume overlaps a non-targeted tumour visible via imaging
22. The targeted tumour(s) is not clearly visible with diagnostic ultrasound and computed tomography (CT) or magnetic resonance (MR) imaging
23. The targeted tumour(s) is located in liver segment 1
24. The Planned Treatment Volume intended to cover the targeted tumour includes or encompasses any portion of the main portal vein, common hepatic duct, common bile duct, gallbladder or stomach/bowel.

### Statistical Analysis

#### Sample Size Calculation

A total of 40 evaluable patients are targeted for enrolment. For sample size estimation, the assumption has been made that one treated lesion per patient. To meet the efficacy objective, there must be ≥ 34/40 (85%) patients achieving Technical Success in the study. With this rule, the Wilson score 95% confidence interval for 34/40 (85%) is (70.9%0.92.9%), and the null hypothesis of a ≤ 70% Technical Success rate (Performance Goal) can be rejected at a 1-sided *p* < 0.025 significance level. By exact binomial probability, the alpha error for this design is 0.024 should the true histotripsy Technical Success rate be 70%. If the true underlying histotripsy Technical Success rate is 90%, the power for observing ≥ 34/40 (85%) successes in the study is approximately 0.90. Due to the allowance of up to 3 treated lesions per patient, the actual 95% confidence interval for the lesion-based Technical Success rate will be estimated by the bootstrap sampling with replacement method and patient will be the sampling unit. The confidence interval will be narrower and the power will be higher than the above estimates. As a 5–10% non-evaluable rate is anticipated, up to 45 patients are targeted for enrolment. In order to minimize bias, a maximum of 20 patients (≤ 50%) may be enrolled at a single site.

#### Evaluation and Trial Enrolment

All patients must be pre-staged with imaging (CT/MRI) at < / = 30 days prior to the planned index procedure. The liver tumours must be well visualied with ultrasound coupled with stand-off ultrasound gel to simulate the treatment water bath- an example would be AQUAFLEX ultrasound gel pad (Parker Laboratories, Inc. Fairfield, NJ, USA). The common barriers for histotripsy treatment are poor acoustic window due to patient’s body habitus hence the tumour is poorly visualized with ultrasound, the tumour is too deep > 15 cm target range or high segment liver tumour location such as segment 7 and 8. All patients are consented as according to the Good Clinical Practice (GCP) code of conduct at the out-patient consultation.

Written site activation from the trial sponsor, including Ethics Committee (EC)/ Institutional Review Board (IRB) approval of the #HOPE4LIVER protocol and Informed Consent Form (ICF) must be obtained prior to enrolling patients in the trial. After the subject has signed the ICF, general inclusion and exclusion criteria have been met, and all imaging criteria have been met, a trial ID number will be assigned and the subject will be considered enrolled in the trial. The trial follow-up period per patient has been extended for 5 years follow-up or until the withdrawal of the subject due to any reason, whichever is first. The trial enrolment process is illustrated in the flow chart.
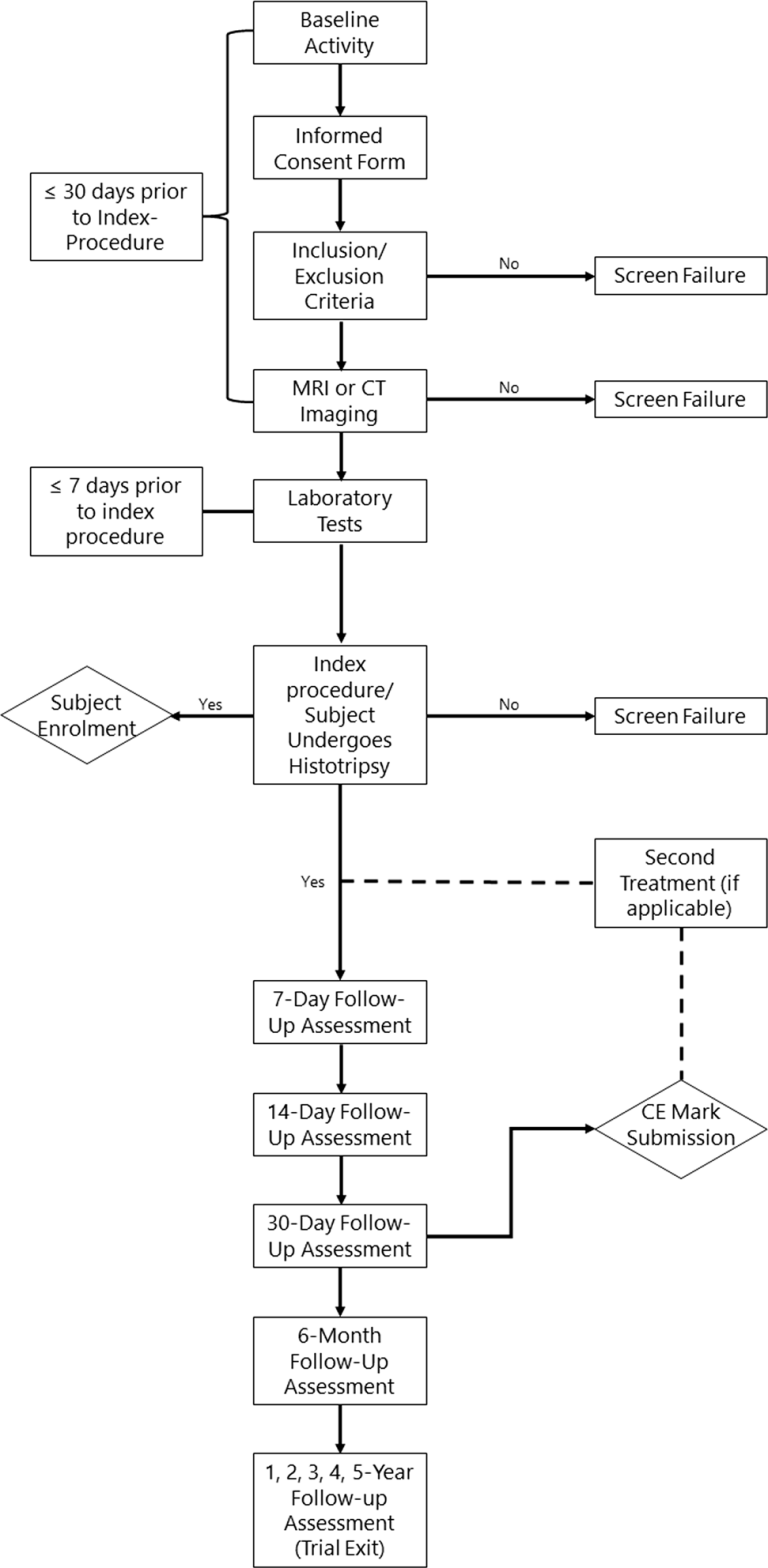


#### Histotripsy Treatment

**Fig. 1:**
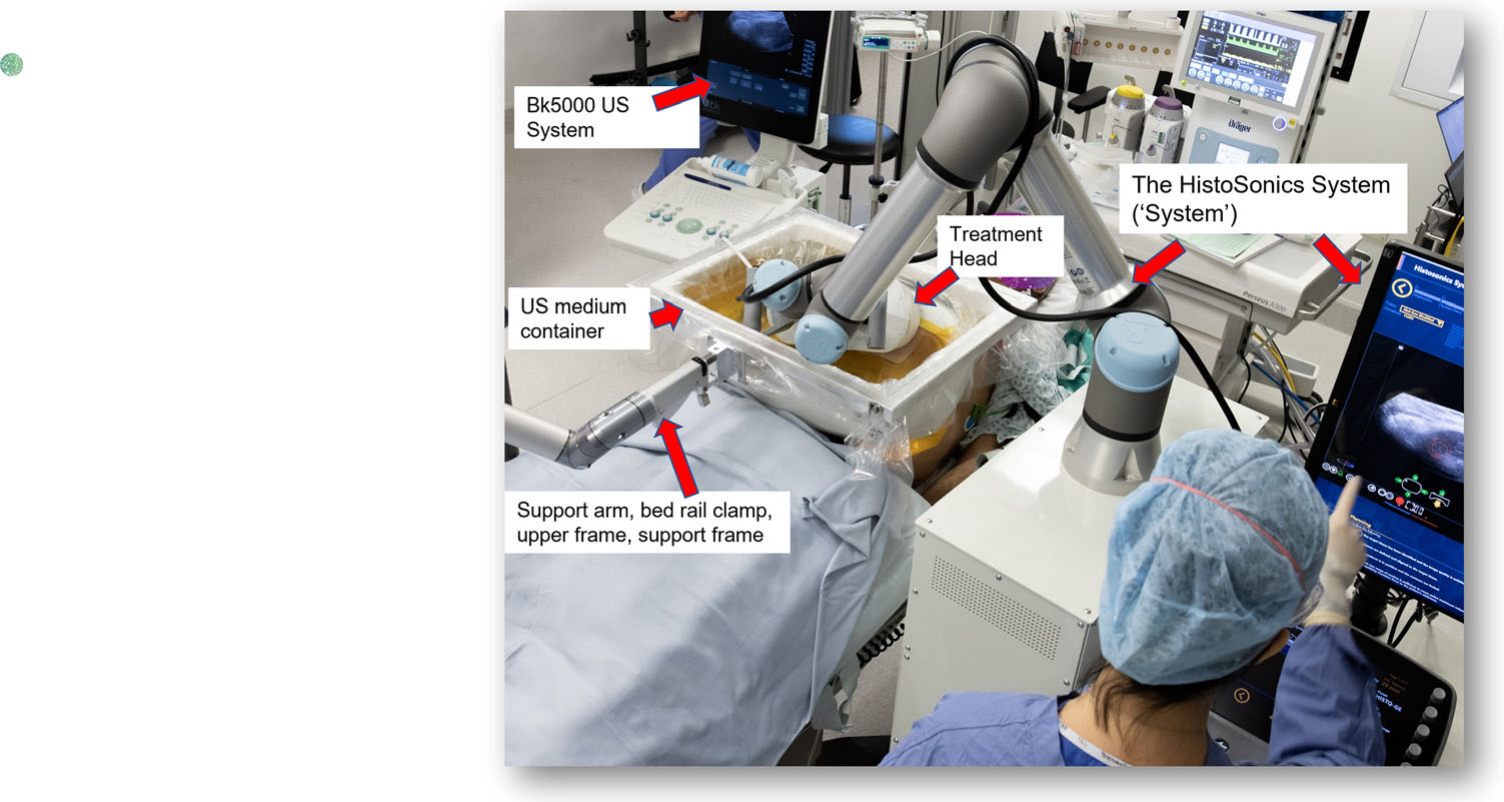
Image guided histotripsy treatment performed under general anaesthesia in interventional radiology suite

This procedure is performed under general anaesthesia. The treatment can be conducted in a theatre or interventional radiology suite (Fig. 1). The procedure is carried out with the trial device provided by the trial Sponsor. For each procedure, a team of engineers will be despatched via HistoSonics to support the treatment procedure and to provide the pre-treatment check for the ‘System’ as necessary. The degassed water bath would be prepared and a trial submersion of the treatment is performed to ensure no bubble formation under the treatment head.

Once the patient is under general anaesthesia, the liver tumour would be re-assessed with ultrasound to ensure visibility and also to locate the treatment window required for the water bath. Once the water is degassed, the treated arm would be navigated through the robotic assisted platform to locate the liver tumour intended for treatment. Once the tumour is targeted, and a required margin is outlined, the required energy for treatment specific to that tissue is determined by creating a bubble cloud at seven predefined locations in the targeted area. These are determined by navigation of the robotic arm through the treatment volume in x, y and z locations. Once the treatment planning is completed, the treatment can be executed automatically by the system with the pre-defined treatment time as stated on the ‘System’.

#### Device Name and Indication for Use

**Fig. 2:**
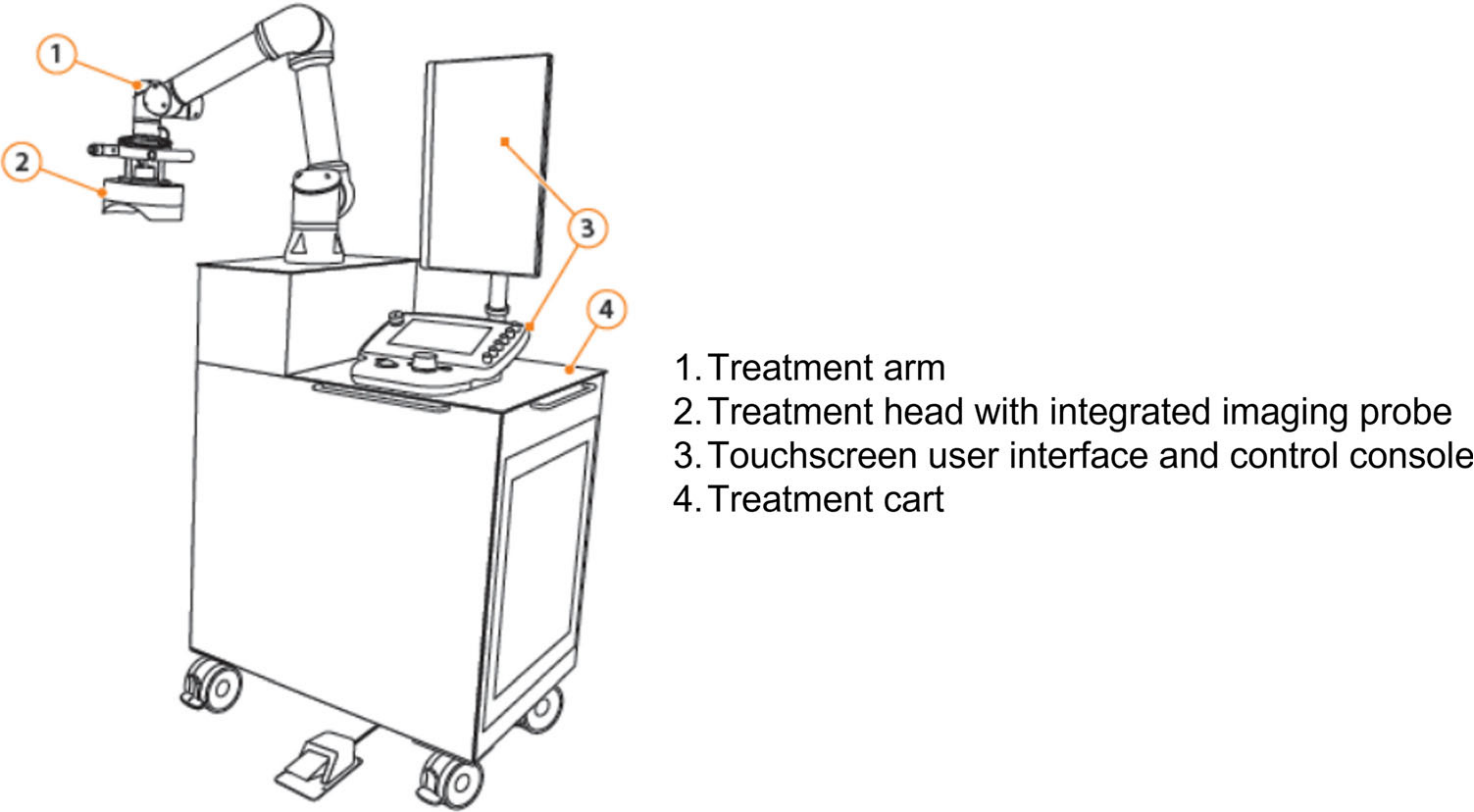
The prototype investigational HistoSonics ‘System’ treatment cart

**Fig. 3:**
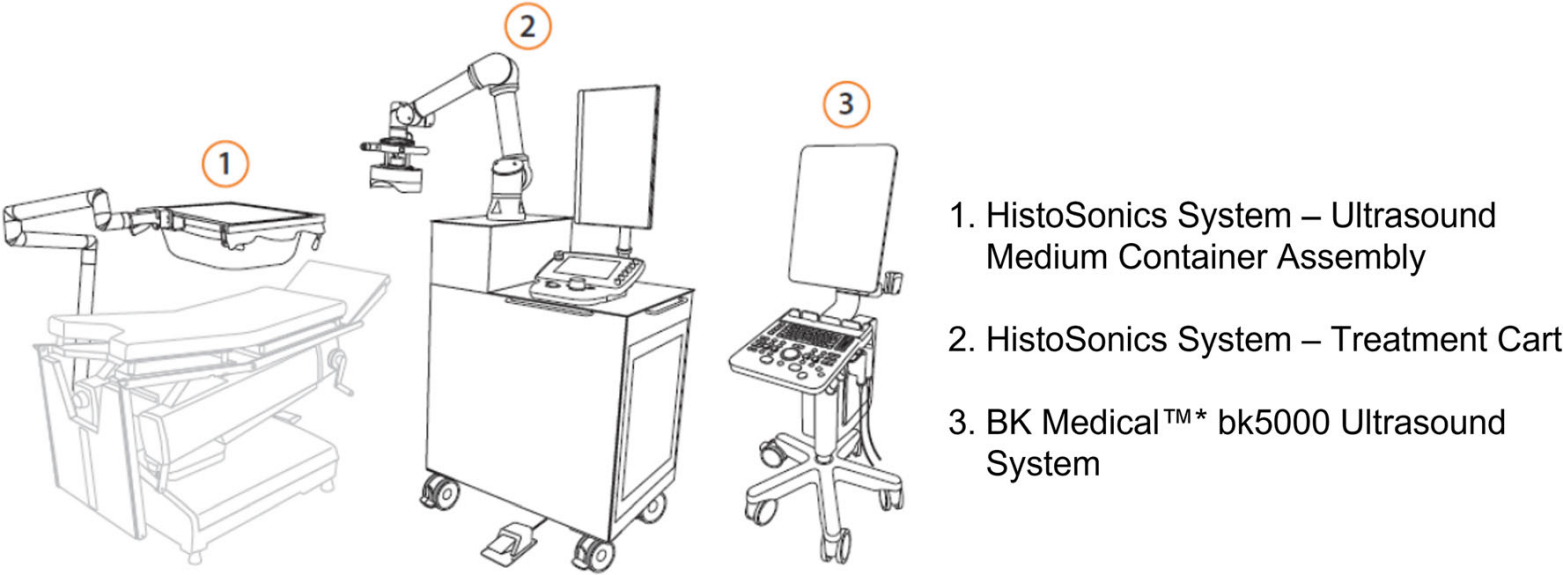
The HistoSonics ‘System’ (ultrasound medium container assembly and treatment cart) and BK Medical ultrasound system

The HistoSonics System (System) (Figs. 2 and 3) is intended for the destruction of liver tissue using histotripsy, a non-thermal, mechanical process using focused ultrasound. The System XFG15261 is manufactured by HistoSonics, Inc. (Plymouth, MN, USA).

Other devices used in the trial have included (Figs. 2 and 3):

- Treatment head 12 cm–XFG15206
- Treatment head, 14 cm–XFG15204
- Bk5000 Ultrasound System–XFG15500
- Support arm, bed rail clamp, upper frame, support frame–XFG15271
- Procedure Kit–XFG80011
  - Ultrasound medium container (single use)
  - Ultrasound medium container drape (single use)
  - Telescoping mirror (single use)
  - Bubble evacuation catheter and syringe (single use)

#### Outcome Measures

The objective of this trial is to evaluate the safety and efficacy of the prototype ‘System’ for the destruction of primary or metastatic tumours located in the liver. For treatment efficacy, the contrast enhanced imaging assessment with CT or MRI is reported as defined by the consensus criteria [32]. The imaging core lab will provide independent review of the imaging assessment to ensure concordance.

Co-Primary outcome measures as defined in the trial protocol [28]:

1. Efficacy: Technical success as determined, at ≤ 36 h post procedure, by evaluating the histotripsy treatment size and coverage. Technical success is defined as the treatment volume/treatment dimensions being greater than or equal to the targeted tumour, and with complete tumour coverage, via CT or MR imaging. For subjects with two treatments, Technical Success will be evaluated at ≤ 36 h post the second procedure.
2. Safety: Procedure-related major complications, defined as CTCAE [31] grade 3 or higher toxicities observed up to 30-days post procedure (second procedure if applies).

The outcomes must be met for both co-primary outcome measures in order for the trial to be considered successful.

Secondary outcome measures:

1. Efficacy: Technique Efficacy defined as the lack of a nodular or mass-like area of enhancement within or along the edge of the treatment volume assessed via CT or MR imaging at 30-days post-procedure (second procedure if applies).
2. Safety: All adverse events reported within 30-days post procedure.

#### Baseline, Pre- and Post-Procedural Assessments

Baseline assessments must be completed ≤ 30 days prior to the planned index-procedure date. A physical exam will be required including but not limited to: height, weight, BMI, temperature, heart rate and respiratory frequency. Pre-procedural assessments must be completed ≤ 7 days prior to the planned start of the index procedure. See Table 1 for baseline, pre-and post-procedural assessment and data collection requirements.

**Table 1:** Summary of required tests and procedures

Procedure/Test/data collection	Baseline	Pre-procedure	Index procedure/ Treatment procedure	Post-procedure/ Discharge	14-Day follow-up phone assessment	30-Day follow-up visit	6-Month follow-up visit	1-Year follow-up visit	2, 3, 4, 5-Year follow-up visit
Visit window	≤ 30 Days prior to index procedure	≤ 7 Days	Procedure	≤ 36 Hours	± 3 Days	± 3 Days	± 30 Days	± 60 Days	± 60 Days
Informed consent	X								
Inclusion/ exclusion criteria	X		X						
Child pugh score	X					X	X	X	X
ECOG PS grade	X					X	X	X	X
Medical history with demographics	X								
Physical exam	X								
Imaging (CT/MRI)	X			X		X	X	X	X
Trial enrolment			X						
Index procedure details			X						
FACT-G	X				X	X	X	X	X
Survey						X			
VAS			X	X					
Technical success				X					
Technique efficacy						X			
End of trial									X
*Clinical laboratory test*									
Blood tests (CBC/ basic metabolic panel/ Liver panel/ tumour biomarkers)		X				X	X	X	X
Pregnancy test (Urine or blood)		X							
*Other*									
Adverse events			X	X	X	X	X	X	X
Complaints/ device deficiencies			X						
Protocol deviations	X	X	X	X	X	X	X	X	X

## Discussion

Histotripsy is based on the delivery of acoustic ultrasound energy in the form of short (< 50 microseconds) very high intensity pulses which induces controlled cavitation to mechanically homogenize targeted tissue [24]. The cavitation occurs when a sufficient negative pressure is applied to the tissue to cause microbubble formation that rapidly expand and collapse producing complete cellular and tissue homogenization. [33]. Specifically, an ultrasound transducer applied external to the body surface through a coupling medium and focused on the intended target area produces highly focal clusters of microbubbles through the delivery of microsecond, high-pressure pulses. Once formed, the microbubbles exhibit highly dynamic patterns of oscillation and inertial collapse which impart severe mechanical stresses on surrounding cells and tissues to produce cellular and tissue destruction at the target area i.e. tumour [17]. In addition, the longer-term response to liver histotripsy treatment was initially investigated in a normal animal model [34], where post histotripsy, majority of the acellular homogenate produced was reabsorbed after 28 days and demonstrated complete tissue resorption within 2 months [35]. In UK, one of the first patients being treated had provided excellent feedback with safe clinical outcome.

This first multi-centre non-randomized phase I/II clinical trial aims to evaluate the initial safety and efficacy of the prototype investigational ‘System’ in the treatment of primary and metastatic liver cancers to support regulatory approval. The study result will be disseminated in peer reviewed scientific publication and provides further insight into future research design.

## References

[1] Adam A, Kenny LM (2015). Interventional oncology in multidisciplinary cancer treatment in the 21(st) century. Nat Rev Clin Oncol.

[2] Zhong J (2017). Cross-sectional study of the provision of interventional oncology services in the UK. BMJ Open.

[3] Reig M (2022). BCLC strategy for prognosis prediction and treatment recommendation: The 2022 update. J Hepatol.

[4] Puijk RS (2018). Colorectal liver metastases: surgery versus thermal ablation (COLLISION)–a phase III single-blind prospective randomized controlled trial. BMC Cancer.

[5] Huang MHJ, Comparison of hepatectomy and local ablation for resectable synchronous and metachronous colorectal liver metastasis (HELARC); 2016. https://clinicaltrials.gov/ct2/show/NCT02886104.

[6] Bruix J, Sherman M (2005). Management of hepatocellular carcinoma. Hepatology.

[7] Solbiati L (2001). Percutaneous radio-frequency ablation of hepatic metastases from colorectal cancer: Long-term results in 117 patients. Radiology.

[8] Dunne RM (2014). Percutaneous treatment of hepatocellular carcinoma in patients with cirrhosis: a comparison of the safety of cryoablation and radiofrequency ablation. Eur J Radiol.

[9] Hu JH (2019). Image-guided percutaneous microwave ablation versus cryoablation for hepatocellular carcinoma in high-risk locations: intermediate-term results. Cancer Manag Res.

[10] Livraghi T (2003). Treatment of focal liver tumors with percutaneous radio-frequency ablation: complications encountered in a multicenter study. Radiology.

[11] Aschoff AJ (2001). How does alteration of hepatic blood flow affect liver perfusion and radiofrequency-induced thermal lesion size in rabbit liver?. J Magn Reson Imaging.

[12] Kudo M (2010). Radiofrequency ablation for hepatocellular carcinoma: updated review in 2010. Oncology.

[13] Mulier S (2005). Local recurrence after hepatic radiofrequency coagulation: multivariate meta-analysis and review of contributing factors. Ann Surg.

[14] Mafeld S (2019). Percutaneous irreversible electroporation (IRE) of hepatic malignancy: a bi-institutional analysis of safety and outcomes. Cardiovasc Interv Radiol.

[15] Zimmerman A, Grand D, Charpentier KP (2017). Irreversible electroporation of hepatocellular carcinoma: patient selection and perspectives. J hepatocell Carcinoma.

[16] Xu Z (2004). Controlled ultrasound tissue erosion. IEEE Trans Ultrason Ferroelectr Freq Control.

[17] Xu Z (2008). Evolution of bubble clouds induced by pulsed cavitational ultrasound therapy–histotripsy. IEEE Trans Ultrason Ferroelectr Freq Control.

[18] Vlaisavljevich E (2013). Image-guided non-invasive ultrasound liver ablation using histotripsy: feasibility study in an in vivo porcine model. Ultrasound Med Biol.

[19] Vlaisavljevich E (2017). Non-invasive liver ablation using histotripsy: preclinical safety study in an in vivo porcine model. Ultrasound Med Biol.

[20] Kim Y (2014). In vivo transcostal histotripsy therapy without aberration correction. Phys Med Biol.

[21] Knott EA (2021). Transcostal histotripsy ablation in an in vivo acute hepatic porcine model. Cardiovasc Interv Radiol.

[22] Longo KC (2019). Robotically assisted sonic therapy (RAST) for noninvasive hepatic ablation in a porcine model: mitigation of body wall damage with a modified pulse sequence. Cardiovasc Interv Radiol.

[23] Xu Z (2021). Introduction to the special issue on histotripsy: approaches, mechanisms, hardware, and applications. IEEE Trans Ultrason Ferroelectr Freq Control.

[24] Parsons JE (2006). Pulsed cavitational ultrasound therapy for controlled tissue homogenization. Ultrasound Med Biol.

[25] Roberts WW (2006). Pulsed cavitational ultrasound: a noninvasive technology for controlled tissue ablation (histotripsy) in the rabbit kidney. J Urol.

[26] Vidal-Jove J (2022). First-in-man histotripsy of hepatic tumors: the Theresa trial, a feasibility study. Int J Hyperthermia.

[27] Chan A-W (2013). SPIRIT 2013 explanation and elaboration: guidance for protocols of clinical trials. BMJ: Br Med J.

[28] ClinicalTrials.gov. The histosonics system for treatment of primary and metastatic liver tumors using histotripsy (#HOPE4LIVER) [cited 2022 10/10]; 2020. https://clinicaltrials.gov/ct2/show/NCT04573881.

[29] Wald C (2013). New OPTN/UNOS policy for liver transplant allocation: standardization of liver imaging, diagnosis, classification, and reporting of hepatocellular carcinoma. Radiology.

[30] ACR. LI-RADS® CT/MRI v2018. [Cited 2022 08/10]; 2018. https://www.acr.org/Clinical-Resources/Reporting-and-Data-Systems/LI-RADS/LI-RADS-CT-MRI--v2018.

[31] NationalCancerInstitute. Common terminology criteria for adverse events (CTCAE). [Cited 2022 08/10]; 2017. https://ctep.cancer.gov/protocoldevelopment/electronic_applications/docs/CTCAE_v5_Quick_Reference_5x7.pdf.

[32] Ahmed M (2014). Image-guided tumor ablation: standardization of terminology and reporting criteria–a 10-year update. J Vasc Interv Radiol.

[33] Maxwell AD (2011). Cavitation clouds created by shock scattering from bubbles during histotripsy. J Acoust Soc Am.

[34] Vlaisavljevich E (2016). Non-invasive ultrasound liver ablation using histotripsy: chronic study in an in vivo rodent model. Ultrasound Med Biol.

[35] Worlikar T (2022). Impact of histotripsy on development of intrahepatic metastases in a rodent liver tumor model. Cancers.

